# Recruitment activities for a nationwide, population-based, group-randomized trial: the VA MI-Plus study

**DOI:** 10.1186/1748-5908-6-105

**Published:** 2011-09-09

**Authors:** Ellen Funkhouser, Deborah A Levine, Joe K Gerald, Thomas K Houston, Nancy K Johnson, Jeroan J Allison, Catarina I Kiefe

**Affiliations:** 1 VA Research Enhancement Award Program (REAP), Birmingham VA Medical Center, Birmingham, AL, USA; 2 Department of Medicine, University of Alabama at Birmingham School of Medicine, Birmingham, AL (DAL adjunct), USA; 3 Ann Arbor VA Healthcare System and Departments of Medicine and Neurology, University of Michigan, Ann Arbor, MI, USA; 4 Veterans Affairs Health Services Research and Development Center of Excellence, Ann Arbor, MI, USA; 5 Community, Environment and Policy, Mel and Enid Zuckerman College of Public Health, University of Arizona, Tucson, Arizona, USA; 6 Department of Quantitative Health Sciences, University of Massachusetts Medical School, Worcester, Massachusetts, USA

## Abstract

**Background:**

The Veterans Health Administration (VHA) oversees the largest integrated healthcare system in the United States. The feasibility of a large-scale, nationwide, group-randomized implementation trial of VHA outpatient practices has not been reported. We describe the recruitment and enrollment of such a trial testing a clinician-directed, Internet-delivered intervention for improving the care of postmyocardial infarction patients with multiple comorbidities.

**Methods:**

With a recruitment goal of 200 eligible community-based outpatient clinics, parent VHA facilities (medical centers) were recruited because they oversee their affiliated clinics and the research conducted there. Eligible facilities had at least four VHA-owned and -operated primary care clinics, an affiliated Institutional Review Board (IRB), and no ongoing, potentially overlapping, quality-improvement study. Between December 2003 and December 2005, in two consecutive phases, we used initial and then intensified recruitment strategies.

**Results:**

Overall, 48 of 66 (73%) eligible facilities were recruited. Of the 219 clinics and 957 clinicians associated with the 48 facilities, 168 (78%) clinics and 401 (42%) clinicians participated. The median time from initial facility contact to clinic enrollment was 222 days, which decreased by over one-third from the first to the second recruitment phase (medians: 323 and 195 days, respectively; *p *< .001), when more structured recruitment with physician recruiters was implemented and a dedicated IRB manager was added to the coordinating center staff.

**Conclusions:**

Large group-randomized trials benefit from having dedicated physician investigators and IRB personnel involved in recruitment. A large-scale, nationally representative, group-randomized trial of community-based clinics is feasible within the VHA or a similar national healthcare system.

## Introduction

Implementation research is the scientific study of methods to promote the rapid uptake of research findings and, hence, improve the health of individuals and populations [1]. Group-randomized trials (GRTs) are an increasingly important tool for implementation research. Typically, individuals (*e.g*., clinicians) are clustered within subunits (*e.g*., clinics) that may be further clustered within higher-level units (*e.g*., facilities or health systems). Accordingly, the unit of randomization and the intervention target may be different (*e.g*., clinics and clinicians, respectively). Unlike the traditional randomized clinical trial (RCT), which focuses on efficacy, implementation research focuses on effectiveness [2,3]. The goal is to understand how efficacious interventions delivered in relatively homogenous populations can be deployed within the community to benefit the population at large. Thus, external validity (generalizability) of GRTs depends on the extent that participants at different levels of clustering represent the population of interest.

Recruitment is important for traditional RCTs, primarily to achieve the needed power to detect significant differences in outcomes; for GRTs, recruitment is important to ensure power and generalizablity. The Myocardial Infarction Plus Comorbidities (MI-Plus) study was a nationwide GRT of Veterans Health Administration (VHA) primary care clinicians who cared for ambulatory post-myocardial infarction (MI) patients, many of whom had multiple comorbidities. The 27-month clinician-directed, Internet-delivered intervention consisted of quarterly case-based interactive educational modules, one to three reviews per month of recently published studies of high clinical impact and relevance to the quality indicators, summaries and links to guidelines applicable to the care of post-MI patients, and downloadable practice tools and patient educational materials [4]. The website was developed using service-oriented architecture and design principles refined in prior studies [5,6]. Iterative usability sessions were used to refine the content. Clinicians in control clinics were provided a link to an existing VHA Office of Quality and Performance website that contained links to a wide range of clinical guidelines for various medical conditions (http://www.healthquality.va.gov/).

Similar to other multicenter implementation studies, the clinic was the unit of randomization [7]. Performance improvement was calculated as the change (pre-intervention period vs. postintervention period) in the proportion of patients receiving each clinical indicator within the clinic [8]. Individual clinicians were embedded within community-based outpatient clinics (clinics), which were embedded organizationally, though not necessarily colocated, within VHA parent facilities (medical centers). This design necessitated several sequential and, at times, simultaneous recruitment efforts targeting individual clinicians, clinics, and facilities. This report describes those recruitment activities as well as the recruitment times and participation rates at the facility, clinic, and clinician level.

## Methods

The VHA is the largest integrated healthcare system in the United States, with 153 medical centers and over 900 ambulatory care and community-based outpatient clinics providing care to an estimated 5.5 million individuals in 2008 [9]. Each facility typically consists of an acute care component, on-site outpatient clinics physically located at the facility, and off-site outpatient clinics distributed across the region served by the facility. Many facilities are also affiliated with an academic medical center and support research activities. Research within the facility must be formally approved by the facility's Institutional Review Board (IRB) and its Research and Development (R&D) committee. Any research conducted at a clinic is governed by the policies of its parent facility.

The study was funded through the VHA Health Services Research and Development (HSR&D) office [IHD 04-387] and by a parallel National Institutes of Health study [R01 HL70786-02][10,11]. We conducted formative work with a panel of expert physicians using nominal group techniques to choose from among 36 potential quality indicators for complex ambulatory post-MI patients that would be both most feasible and most valid [8]. We also conducted focus groups and case-vignette surveys of clinicians, including VHA clinicians, to develop the intervention. The Birmingham VA Medical Center, Birmingham, AL, served as the study's coordinating center. After approval from its IRB and R&D committees, the Birmingham facility and its six affiliated outpatient clinics were the first study enrollees in November 2003.

A priori, we planned a sample size of 200 clinics to provide > 80% statistical power to detect a 5% difference in improvement between intervention and control clinics for all of the primary clinical indicators over a range of assumptions. Our initial recruitment plan allotted six months to recruit the 200 clinics using a strategic approach of recruiting parent facilities using high-yield targets (*i.e*., personal contacts) and leveraging regional leadership support for our study. As only one-third of the requisite clinics were recruited after eight months, we re-evaluated the initial recruitment procedures (phase 1) and revised them to improve recruitment in phase 2.

### Phase 1 facility recruitment protocol

In the first phase of recruitment (April 2004-November 2004), potential facilities for recruitment were identified using the 2003 VA Station Tracking (VAST) database. To be eligible, facilities had to have an affiliated IRB; four or more eligible clinics; and no ongoing, potentially overlapping, quality-improvement project. The four-clinic requirement was relaxed towards the end of recruitment. Clinics were eligible if they were noncontract (owned and operated by VHA), delivered primary care, used the VHA's electronic health record (EHR) system, and provided Internet access to all clinicians. For eligible facilities, we sought a physician willing to serve as a local principal investigator (PI). To identify funded investigators and other potential contacts within each facility who could serve as a local PI, the VHA R&D and HSR&D websites were reviewed. The subsequent list was reviewed by our study investigators to identify high-yield targets (*i.e*., personal contacts) to initiate facility recruitment. The project coordinator (a registered nurse) called and emailed these high-yield targets on behalf of the study investigators.

### Phase 2 facility recruitment protocol

**Figure 1:**
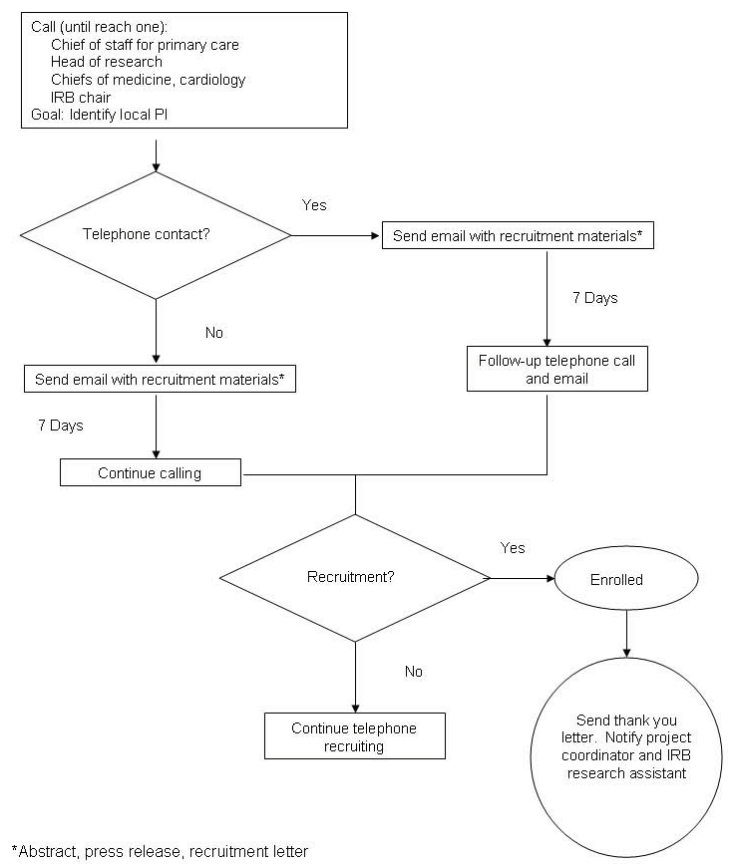
**Facility recruitment scheme (phase 2): the VA MI-Plus study**.

**Figure 2:**
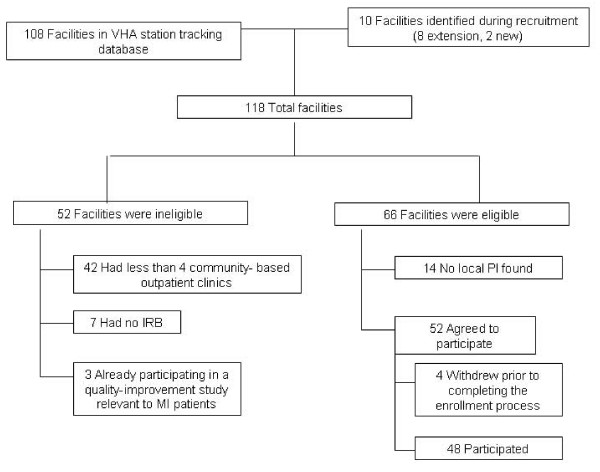
**Facility participation: the VA MI-Plus study**.

In the second phase of facility recruitment (December 2004-July 2005), "cold" contacts were recruited via emails and telephone by targeting facilities with the largest number of associated clinics. To increase recruitment and its efficiency, we developed and implemented a standardized recruitment protocol (Figure 1). During this second phase, we added two physician investigators to assume primary responsibility for recruitment of local PIs and leverage physician-to-physician communications [12]. To facilitate the IRB approval process at participating facilities, a research assistant was also hired to specifically oversee all IRB protocols and to prepare a standard IRB packet for each facility. In phase 1, research staff assisted each facility with IRB preparation but did not prepare a standard IRB packet. The 14 facilities for which a local PI could not be found or for which IRB approval was never obtained were classified as "declined" facilities (Figure 2).

The physician investigators followed the recruitment protocol shown in Figure 1. Contact information (name, position, telephone number, and email) for potential physician PIs was obtained from the VAST database and facility websites. These initial emails contained recruitment materials (the study abstract, a press release, and a recruitment letter), outlined the need for a local PI, and described the general expectations of this position. During the telephone call, questions were answered, interest was ascertained, and if the individual declined to participate, they were asked to refer others who might be interested. This process was continued until a local PI was identified or all leads were exhausted, including contacting the chief of medicine, IRB chair, and chief of staff. To cover costs of participation, the facility received a site distribution of $2,500.

Facilities were recruited until we achieved our goal of 200 eligible clinics. While facility recruitment continued in a rolling fashion, we simultaneously recruited clinics and clinicians of enrolled facilities to participate in the intervention study. Clinicians at each facility's associated clinics were not recruited or provided study materials until a local PI was identified, all IRB requirements were met, and a list of all eligible clinicians and their email addresses were obtained. The date these materials were approved and posted was the facility's launch date.

### Clinic and clinician recruitment protocol

A clinic was enrolled and randomized when the first eligible clinician (a physician, physician assistant, or nurse practitioner) at that clinic logged on to the study website. All clinicians at a clinic were randomized to the same arm, but only clinicians who logged on were enrolled. Clinicians were recruited continuously throughout the two-year intervention period. Immediately following the facility launch, clinicians were sent an email, a postal letter, and study flier that described the study and how to log on to the study webpage. Subsequent weekly email and fax reminders were sent to clinicians who had not yet logged on. Approximately four to six weeks after the facility launch date, one of the study physicians sent a more personalized email to each clinician at clinics not yet enrolled (this involved seven clinics over the course of the study). If unsuccessful, telephone contact was attempted with each clinician at clinics not yet enrolled. Telephone attempts were discontinued if a clinician was reached or three attempts were made. The date and type of contact attempt was tracked in an Excel (Microsoft Corporation, Redmond, WA, USA) spreadsheet; however, more recent contact attempts were overwritten on earlier attempts. The primary goal of these attempts was to increase clinic enrollment and not clinician enrollment (*i.e*., if any clinician at a given clinic logged on, the clinic was considered to be enrolled). Proactive emails were sent notifying all clinicians (enrolled or nonenrolled) of new updates and materials. Such reminders have been demonstrated to increase participation in Internet-delivered clinician interventions [13]. Lastly, a monthly recruitment report was emailed to the local PI at the associated parent facility. The report contained the name of each clinician and his/her enrollment status. Local PIs were encouraged to informally facilitate recruitment where feasible by encouraging their peers to log on. All enrolled intervention and control clinicians could obtain continuing education credits for reviewing eligible educational materials on the website. No other incentives were provided owing to VHA policy.

### Statistical analysis

Differences in facility participation rates were assessed according to the presence of a formally funded existing VHA HSR&D program at the time of recruitment (defined as a Center of Excellence, Research Enhancement Award Program, or a Targeted Research Enhancement Program), rural-urban locale,[14] geographic region of the United States, and facility size in terms of number of affiliated clinics. Differences in clinic participation rates among participating facilities were similarly assessed, with clinic size classified according to the number of affiliated clinicians. The analyses were repeated among participating facilities to assess differences by recruitment phase.

We defined four time intervals to represent the different aspects of the total recruitment time for a clinic: (1) initial facility contact to recruitment of a local PI, (2) recruitment of a local PI to approval by both the R&D committee and the IRB, (3) IRB or R&D approval (whichever the facility required last) to launch, and 4) launch to first clinic enrollment. Interval 2 represents an estimate of facility approval time. Because all time intervals were skewed, the median was used as a measure of central tendency. Kruskal-Wallis tests were used to assess differences of time variables across categorical variables, and Spearman rank correlations were used to measure associations of selected characteristics, specifically, measures of facility and clinic size, with time intervals.

## Results

### Facility participation

**Figure 3:**
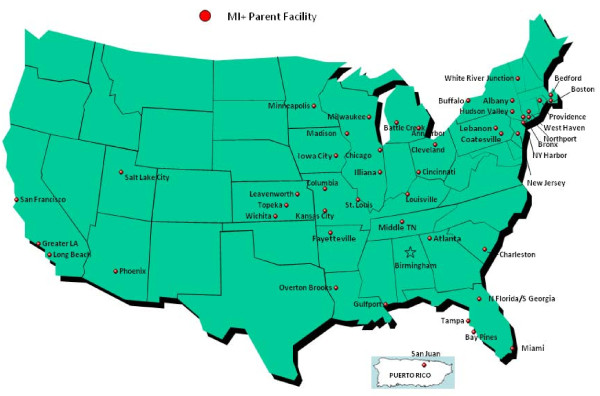
**Geographic locations of participating facilities: the VA MI-Plus study**.

Of 118 facilities identified as potentially eligible (Figure 2), 66 were confirmed eligible. Of the 66, 12 had a formally established VA HSR&D program and 59 were in an urban area (Table 1). The largest proportion (n = 23, 35%) was in the midwest and the lowest in the west (n = 12, 18%). The median number of affiliated clinics was five (interquartile range of four to six). Forty-eight facilities (73%) located across the continental United States (Figure 3) participated. Facility participation rates were higher among facilities with a formally established VA HSR&D program, those located in an urban area, those not in the west, and those that were smaller (Table 1).

**Table 1:** Distribution of eligible facilities according to participation: the VA MI-Plus study

			Participated				
Characteristics	ALL (N = 66)		YES (N = 48)		NO (N = 18)		*p*
	**N**	**%**	**N**	**%**	**N**	**%**	
Had a VHA health services research program	12	18.2	12	25.0	0	0.0	.03
Located in an urban area	59	89.4	45	95.7	14	77.8	.045
Geographic region							.03
New England/Mid-Atlantic	16	24.2	14	29.2	2	11.1	
Midwest	23	34.8	16	33.3	7	38.9	
South	15	22.7	13	27.1	2	11.1	
West	12	18.2	5	10.4	7	38.9	
Number of affiliated outpatient clinics							.07*
2-3	16	24.2	14	29.2	2	11.1	
4-5	30	45.4	22	45.8	8	44.4	
6-15	20	30.3	12	25.0	8	44.4	
Median (interquartile range)	4.5	(4-6)	4	(3-5.5)	5	(4-7)	.02**

*p for trend test; **p for Wilcoxon rank sum test.

VHA = Veterans Health Administration.

For the 18 facilities that did not participate, a willing PI could not be identified in 14 (Figure 2), with two facilities citing serious staffing problem/staffing turnover as reasons. Willing PIs were found at another 4 of the 18 facilities but their research offices declined for two of them, and another two never completed the IRB approval process.

### Clinic participation

There were 219 clinics affiliated with the 48 participating facilities, of which 168 (77%) participated (Table 2). As with facilities, most clinics were located in urban areas, with relatively few in the west. The median clinic size (number of clinicians) was 3 (range 1 to 15). Larger clinics and clinics located in urban areas were more likely to participate than their counterparts; clinic participation did not differ by US region.

**Table 2:** Distribution of community-based outpatient clinics and associated clinicians among the 48 participating facilities: the VA MI-Plus study

	Clinics			Clinicians		
	
	Total	Participating		Total	Participating	
	**N**	**N**	**%**	**N**	**N**	**%**
ALL	219	168	76.7%	957	401	41.9%
Located in an urban area^a^						
Yes	171	139	81.3%	828	350	42.3%
No	42	26	61.9%	100	44	44.0%
			*p = .007*			*p = .7*
Geographic region						
New England/Mid-Atlantic	62	48	77.4%	199	98	49.2%
Midwest	70	55	78.6%	273	117	42.9%
South	63	48	76.2%	373	145	38.9%
West	18	14	77.8%	83	34	41.0%
Puerto Rico & Virgin Islands	6	3	50.0%	29	7	24.1%
			*p = .6*			*p = .046*
Number of clinicians						
1	38	18	47.4%	39	17	43.6%
2-3	93	71	76.3%	227	107	47.1%
≥ 4	88	79	89.8%	691	277	40.1%
			*p < .001*			*p = .2*

^a^Does not include the six clinics and 29 clinicians from Puerto Rico & the Virgin Islands.

### Clinician participation

There were 957 clinicians affiliated with the 219 clinics, of whom 401 (42%) participated (Table 2). In contrast to clinic participation rates, clinician participation rates did not differ by rural-urban locale or clinic size. As with clinics, clinician participation rates did not differ by geographic region within the continental United States.

### Facility recruitment time

**Figure 4:**
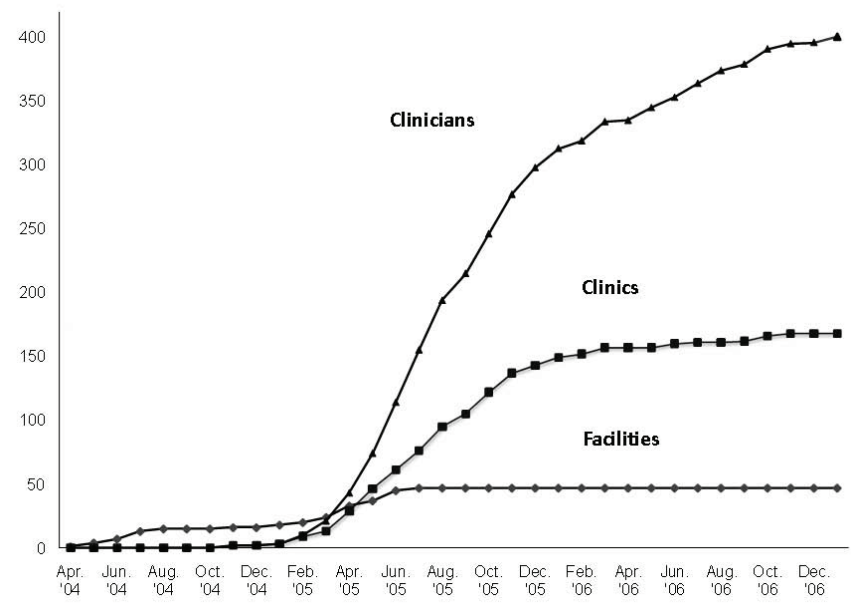
**Cumulative enrollment by month: the VA MI-Plus study**.

**Figure 5:**
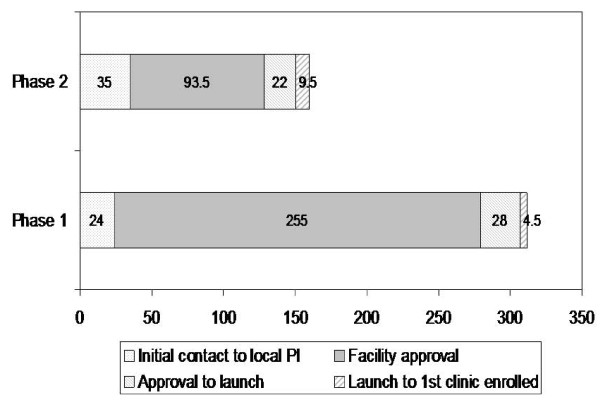
**Median number of days for component intervals from initial facility contact to first clinic enrollment: the VA MI-Plus study**.

Excluding the coordinating center, the facilities were recruited over a 15-month period. Between April 2004 and November 2004, 16 facilities (25% of 65 eligible) were recruited, and between December 2004 and July 2005, 31 facilities were recruited (63% of 49 remaining eligible) (Figure 4). The median time from initial facility contact to clinic enrollment was 222 days. This interval decreased by over a third, from a median of 323 to 195 days (*p *< .001), from the first to second recruitment phase (Figure 5). This was largely due to a decrease in facility approval time (255 to 94 days; *p *< .001), which remained the largest component of recruitment time. In all but three facilities, approval by the R&D committee was required before submission for IRB approval. This initial approval constituted 80% (93 of 116 days) of overall median facility approval time, a percent similar for both phases of the study. Initial contact time (*i.e*., time to identification of local PI) did not differ by facility size overall (*r *= .01) or in either phase. Facility approval time was associated with facility size in the first phase (*r *= .68) but not the second phase (*r *= -.01). As expected, clinic size was inversely (*r *= -.51) associated with time from facility launch date to clinic enrollment (participation); namely, clinics with more clinicians had a clinician log on sooner than did clinics with fewer clinicians, and this association was present in both recruitment phases of the study.

### Clinic/clinician recruitment time

Over half (n = 90; 53%) of the clinics enrolled within one week of facility launch and most (n = 146; 87%) enrolled within four weeks. Six weeks after facility launch, only 7% of clinics had not enrolled. This pattern was the same for both recruitment phases. The longest time period to enroll a clinic was 10 weeks (n = 3 clinics). Regarding time to last new clinician logging on within a clinic, 25% of clinics had the last new clinician participate by four weeks, 50% by 7.5 weeks, and 75% by 28 weeks. One clinic had a clinician who first logged on 80 weeks after initial invitation. Although we did not formally gather information on clinician refusal, which was passive, qualitatively, most clinicians who did not enroll and were reached by telephone cited lack of time and interest as reason for not participating.

## Discussion

The possibility of obtaining a large, nationally representative sample of primary care clinicians (physicians, physician's assistants, and nurse practitioners) makes the VHA health system an enticing setting to conduct implementation and outcomes research. With careful planning, a systematic yet flexible approach, and a multidisciplinary staff, it is possible to recruit a nationwide sample of primary care clinicians employed in the VHA's community-based outpatient clinics. Over approximately two years, we were able to recruit 401 clinicians representing 168 clinics and 48 facilities in 26 states and Puerto Rico and the Virgin Islands. These groups accounted for 73% of all eligible facilities, over 75% of their associated clinics, and 42% of their clinicians.

Most GRTs do not report a response rate as they have a target number of "groups" or practices to recruit for the purposes of statistical power [15] and do not identify, or at least report, a sampling denominator. Our facility and clinic response rates were much higher than the 27% of nursing homes in a GRT study of osteoporosis fracture prevention [16] or the 33% of practices in a managed-care organization's study to increase chlamydia screening [5]. Our response rate is similar to non-GRT studies where the purpose was to obtain a population-based nationwide sample. For example, the National Institutes of Health-funded Coronary Artery Risk Development in Young Adults (CARDIA) study has been following an initial cohort of 5,115 community-dwelling healthy young adults first recruited in 1985 for nearly 25 years. The initial 1985 recruitment for CARDIA resulted in a 55% response rate [17]. CARDIA has significantly contributed to our scientific knoweldge, having resulted in over 400 peer-reviewed publications. More recently, the National Institues of Health-funded Cancer Outcomes Research Consortium (CanCORS) was established in 2001 to obtain a representative, population-based sample to study the processes and outcomes of patients with newly diagnosed lung or colorectal cancer [18]. The approximately 10,000 cancer patients recruited with a population-based approach represent about 50% of the underlying target population. As the recruitment methodologies of GRTs become more refined, their findings will be highly generalizable.

Recruiting for GRTs and for RCTs can be viewed under similar theoretical perspectives, including Choo's general model of information use identifying major elements that influence information-seeking behavior [19] and the work of Christensen and Armstrong involving diffusion of innovation [20], which includes "disruptive" effects. In the VA MI-Plus study, recruitment involved two groups of clinicians: (1) physicians to identify a local PI and (2) clinicians to log on and participate in the intervention. These clinician groups may have different elements that influence their participation. Local PIs had to complete necessary IRB training and submit applications through R&D and IRB committees for study approval at their facility. Even with the parent site (Birmingham) preparing necessary packages in the second phase, obtaining these approvals could be quite time consuming. There was no direct compensation to these individuals. Reasons to participate, as cited by another GRT [21], may include the desire to improve their clinical practice or an interest in contributing to medical knowledge in general, but these benefits must exceed any perceived disruptive effects. In comparison, at the clinic level, a clinician simply had to log on to enroll and thus be classified as participating.

In comparing phase 1 and phase 2 recruiting, we found, as have others [21,22], that physician-to-physician recruiting gave a much greater yield and that prior personal contacts did not have a substantial effect. We also learned that recruitment strategies needed to change over time in order to achieve recruitment targets. Similarly, Ellis *et al*. [21] used 10 different nonrandomized strategies over 11 months to recruit sufficient practices in the GLAD HEART study, a total of 61 practices, all within one US state. In a review of recruitment rates and strategies across studies conducted in one medical center, Johnston *et al*. [23] found considerable variation in recruitment rates despite similar strategies and staffing. Number of recruited practices ranged from 30 to 137; most required over nine months to recruit and most had not planned for the time needed. They found personal connections helpful and have suggested that these personal connections can be developed during the recruiting process. We also found that buy-in from participants (the use of local PIs to champion the study) and a flexible recruitment strategy enhanced recruitment, findings consistent with those of Johnston et al. [23]

Minimization of possible disruptive effects for the clinician may have facilitated recruitment in our study. First, VHA's use of EHRs made it possible to extract patient records without interfering with office flow. Also, randomization and analysis was at the clinic level, thus low-performing individual clinicians were not at risk of being identified. Similarly, the use of EHRs and clinics as the randomization unit enabled the recruitment of 20 practices in 14 states for a multimethod GRT [24]. The Ornstein study relied on academic detailing and site visits, components that may be disruptive from the theoretical perspective and expensive or impractical for a nationwide study. Interestingly, the parallel MI-Plus study involving primary care clinicians in Alabama and Mississippi [25,10,8] had a much lower participation rate (13%) for clinicians [25], perhaps because these clinicians lacked EHRs and viewed manual chart abstraction as disruptive to their practices.

Between the first and second phases of our recruitment, the amount of time required to obtain facility approval of the study protocol decreased from a median of 255 days to 94 days. This 63% reduction was primarily attributed to the addition of an experienced IRB staff member at the Study Coordinating Center that allowed for the implementation of a more systematic and structured approach to IRB management. The complexity and sheer volume of work needed to coordinate IRB approval for 48 participating facilities cannot be overstated. The majority of facilities required R&D approval prior to IRB submission, and obtaining R&D approval constituted the bulk of the facility approval time, with IRB approval requiring only an additional two to four weeks. This may be misleading in that many R&D committees wanted "the essence of the IRB packet" to review, thus, an IRB specialist is invaluable in facilitating R&D approval as well.

Establishment of the recently implemented central IRB in the VHA (an IRB approved by a central office to cover all participating facilities in a multisite study) should enhance the efficiency, cost, and attractiveness of conducting nationwide GRTs within the VHA. Use of single-study IRB cooperative agreements in the (beta)-Carotene and Retinol Efficacy Trial (CARET) in a university setting reduced the average time to complete IRB approval from over six months to one month for each of many substudies [26]. Even with a central IRB, we anticipate, as have others [27-29], that a dedicated research assistant or IRB specialist is advised in the planning of any large GRT within or external to the VHA. In 2005, with an established protocol and experienced staff, it took approximately six months from initial contact at a facility to enroll an associated clinic; half of this time (three months) was for facility approval, which perhaps can be reduced to one month with the central IRB recently implemented by the VHA. One challenge that will remain, even with a central IRB, is getting PIs to do requisite training in research practices (*e.g*., good clinical practices, privacy, and security training) needed for IRB approval. This required substantial effort from our study staff, primarily that of the IRB specialist. In an era of ever-increasing regulatory oversight, we believe that this will persist as a substantial task that should be planned for when designing studies and budgeting personnel. A database of and for VHA researchers to register and complete the approval and training necessary to do VHA research should facilitate the recruitment process.

Our conclusions regarding the importance of a functional, truly interdependent relationship between the study PI and the clinical research coordinator echo those of other teams [30]. The success of our study would not have been possible without a close collaboration between these two members of the research team. Evaluating the value-added contribution of such a position should be an important future consideration.

Our experience suggests that using a recruitment approach that seems counterintuitive might be warranted. Our initial efforts to recruit local PIs focused on high-yield targets (*i.e*., personal contacts), largely due to initial anxiety on the part of the recruitment team of cold calling. While recruiting based on familiarity might have made us feel better, the cold peer-to-peer calling successfully recruited many local PIs and proved less difficult and more efficient than anticipated. We might have saved time and improved study efficiency by expending more energy on cold calling local PIs early and getting the recruitment process started and saving the "easy" recruits for later. Anecdotally, cold calling individual clinicians to log on was not nearly as successful a recruitment tool as cold calling for local PIs. This observation may be a result of being able to offer the facility of local PIs a site distribution of funds ($2,500) to cover costs of participating, while we could not offer clinicians any similar distribution of funds for participation in the study owing to VHA policy.

## Conclusions

We found that having dedicated research team members, physician investigators, and an IRB specialist actively involved in the recruitment process and using a standardized recruitment protocol greatly increased the ability and efficiency of facility recruitment. These specialized personnel, however, appeared to have very little effect on recruiting clinics and clinicians. We believe that our study demonstrates the ability to do implementation research with a level of generalizability comparable to that of major epidemiologic studies. As group-randomized implementation trials become more common, large healthcare systems, such as the VHA, will provide us with the opportunity to refine our methods and become key "laboratories" for the development of implementation science.

## Competing interests

This project was funded in part by grant SDR 03-090-1 from the VA Health Services Research and Development (HSR&D) and by grant number R01 HL70786 from the National Heart, Lung, and Blood Institute.

## Authors' contributions

All authors reviewed drafts of the paper and read and approved the final version. EF performed all analyses and drafted the paper. DAL was a study investigator and developed the protocol for recruiting physicians and the intervention content. JKG was a study investigator who personally assisted in recruiting physicians. TKH was a study investigator who led the design of the Internet intervention. NKJ was a study coordinator who personally assisted in recruiting and tracking physicians. JJA was a study investigator who advised on study design, especially regarding implementation. CIK conceived the overall design of the study and oversaw all aspects of the study.

## References

[B1] Kiefe CI Sales A A state-of-the-art conference on implementing evidence in health care. Reasons and recommendations J Gen Intern Med 2006 21 Suppl 2 S67 70 16637964 10.1111/j.1525-1497.2006.00366.x PMC2557139

[B2] Glasgow RE Emmons KM How can we increase translation of research into practice? Types of evidence needed Annu Rev Public Health 2007 28 413 433 10.1146/annurev.publhealth.28.021406.144145 17150029

[B3] Salanitro A Estrada C Allison J Glasser S Implementation research: beyond the traditinal randomized controlled trial Essentials of Clinical Research 2008 New York, NY: Springer and Associates 217 244

[B4] Houston TK Funkhouser EM Levine DA Allison JJ Williams OD Kiefe CI Developing measures for provider participation in internet delivered interventions: Comparison of three randomized trials MedInfo 2007 12 2 4 17911944

[B5] Allison JJ Kiefe CI Wall T Casebeer L Ray MN Spettell CM Hook EW Oh MK Person SD Weissman NW Multicomponent Internet continuing medical education to promote chlamydia screening Am J Prev Med 2005 28 3 285 290 10.1016/j.amepre.2004.12.013 15766617

[B6] Houston TK Funkhouser E Allison JJ Levine DA Williams OD Kiefe CI Multiple measures of provider participation in Internet delivered interventions Stud Health Technol Inform 2007 129 Pt 2 1401 1405 17911944

[B7] Glynn RJ Brookhart MA Stedman M Avorn J Solomon DH Design of cluster-randomized trials of quality improvement interventions aimed at medical care providers Med Care 2007 45 10 Supl 2 S38 43 17909381 10.1097/MLR.0b013e318070c0a0

[B8] Pena A Virk SS Shewchuk RM Allison JJ Williams OD Kiefe CI Validity versus feasibility for quality of care indicators: expert panel results from the MI-Plus study Int J Qual Health Care 2010 22 3 201 209 10.1093/intqhc/mzq018 20382663 PMC2868528

[B9] 2008 VA Sheet Fact http://www.va.gov/health/MedicalCenters.asp Accessed April 5, 2011

[B10] Sales AE Tipton EF Levine DA Houston TK Kim Y Allison J Kiefe CI Are co-morbidities associated with guideline adherence? The MI-Plus study of Medicare patients J Gen Intern Med 2009 24 11 1205 1210 10.1007/s11606-009-1096-4 19727967 PMC2771234

[B11] Funkhouser E Houston TK Levine DA Richman J Allison JJ Kiefe CI Physician and patient influences on provider performance: beta-blockers in postmyocardial infarction management in the MI-Plus study Circ Cardiovasc Qual Outcomes 2011 4 1 99 106 10.1161/CIRCOUTCOMES.110.942318 21139090 PMC3099457

[B12] Greene SM Geiger AM A review finds that multicenter studies face substantial challenges but strategies exist to achieve Institutional Review Board approval J Clin Epidemiol 2006 59 8 784 790 10.1016/j.jclinepi.2005.11.018 16828670

[B13] Houston TK Coley HL Sadasivam RS Ray MN Williams JH Allison JJ Gilbert GH Kiefe CI Kohler C Impact of content-specific email reminders on provider participation in an online intervention: a dental PBRN study Stud Health Technol Inform 2010 160 Pt 2 801 805 20841796 PMC2967030

[B14] Measuring Rurality: Rural-Urban Commuting Area Codes US Department of Agriculture, Economic Research Service Update date: September 2, 2005

[B15] Houston TK Richman JS Ray MN Allison JJ Gilbert GH Shewchuk RM Kohler CL Kiefe CI Internet delivered support for tobacco control in dental practice: randomized controlled trial J Med Internet Res 2008 10 5 e38 10.2196/jmir.1095 18984559 PMC2630831

[B16] Colon-Emeric CS Lyles KW House P Levine DA Schenck AP Allison J Gorospe J Fermazin M Oliver K Curtis JR Randomized trial to improve fracture prevention in nursing home residents Am J Med 2007 120 10 886 892 10.1016/j.amjmed.2007.04.020 17904460 PMC2288656

[B17] Friedman GD Cutter GR Donahue RP Hughes GH Hulley SB Jacobs DR Liu K Savage PJ CARDIA: study design, recruitment, and some characteristics of the examined subjects J Clin Epidemiol 1988 41 11 1105 1116 10.1016/0895-4356(88)90080-7 3204420

[B18] Ayanian JZ Chrischilles EA Fletcher RH Fouad MN Harrington DP Kahn KL Kiefe CI Lipscomb J Malin JL Potosky AL Understanding cancer treatment and outcomes: the Cancer Care Outcomes Research and Surveillance Consortium J Clin Oncol 2004 22 15 2992 2996 10.1200/JCO.2004.06.020 15284250

[B19] Choo CW The knowing organization: How organizations use information to construct meaning, create knowledge, and make decisions 2005 Second New York: Oxford University Press

[B20] Christensen CM Armstrong EG Disruptive Technologies: a credible threat to leading programs in continuing medical education? Journal of Continuing Education in the Health Professions 1998 18 2 69 80 10.1002/chp.1340180202

[B21] Ellis SD Bertoni AG Bonds DE Clinch CR Balasubramanyam A Blackwell C Chen H Lischke M Goff DC Jr Value of recruitment strategies used in a primary care practice-based trial Contemp Clin Trials 2007 28 3 258 267 10.1016/j.cct.2006.08.009 17030154 PMC3760001

[B22] Bertoni AG Bonds DE Chen H Hogan P Crago L Rosenberger E Barham AH Clinch CR Goff DC Jr Impact of a multifaceted intervention on cholesterol management in primary care practices: guideline adherence for heart health randomized trial Arch Intern Med 2009 169 7 678 686 10.1001/archinternmed.2009.44 19364997 PMC2937279

[B23] Johnston S Liddy C Hogg W Donskov M Russell G Gyorfi-Dyke E Barriers and facilitators to recruitment of physicians and practices for primary care health services research at one centre BMC Med Res Methodol 2010 10 109 10.1186/1471-2288-10-109 21144048 PMC3017524

[B24] Ornstein S Jenkins RG Nietert PJ Feifer C Roylance LF Nemeth L Corley S Dickerson L Bradford WD Litvin C A multimethod quality improvement intervention to improve preventive cardiovascular care: a cluster randomized trial Ann Intern Med 2004 141 7 523 532 15466769 10.7326/0003-4819-141-7-200410050-00008

[B25] Schoen M Tipton EF Houston TK Funkhouser E Levine DA Estrada C Allison J Williams OD Kiefe CI Characteristics that predict physician participation in a web-based CME activity: The MI-Plus study (NHLBI MI+) Continuing Edcuation in the Health Professions 2009 29 4 246 253 10.1002/chp.20043 PMC3155512 19998447

[B26] Thornquist MD Edelstein C Goodman GE Omenn GS Streamlining IRB review in multisite trials through single-study IRB Cooperative Agreements: experience of the Beta-Carotene and Retinol Efficacy Trial (CARET) Control Clin Trials 2002 23 1 80 86 10.1016/S0197-2456(01)00187-8 11852169

[B27] Dziak K Anderson R Sevick MA Weisman CS Levine DW Scholle SH Variations among Institutional Review Board reviews in a multisite health services research study Health Serv Res 2005 40 1 279 290 10.1111/j.1475-6773.2005.00353.x 15663713 PMC1361137

[B28] Green LA Lowery JC Kowalski CP Wyszewianski L Impact of institutional review board practice variation on observational health services research Health Serv Res 2006 41 1 214 230 10.1111/j.1475-6773.2005.00458.x 16430608 PMC1681539

[B29] Vick CC Finan KR Kiefe C Neumayer L Hawn MT Variation in Institutional Review processes for a multisite observational study Am J Surg 2005 190 5 805 809 10.1016/j.amjsurg.2005.07.024 16226962

[B30] Pelke S Easa D The role of the clinical research coordinator in multicenter clinical trials J Obstet Gynecol Neonatal Nurs 1997 26 3 279 285 10.1111/j.1552-6909.1997.tb02143.x 9170591

